# Drivers of strong isolation and small effective population size at a leading range edge of a widespread plant

**DOI:** 10.1038/s41437-023-00610-z

**Published:** 2023-04-04

**Authors:** Anita Cisternas-Fuentes, Matthew H. Koski

**Affiliations:** Department of Biological Sciences, Clemson University, 132 Long Hall, Clemson, SC 29634 USA

**Keywords:** Genetic variation, Evolutionary ecology

## Abstract

Climate change has influenced species distributions worldwide with upward elevational shifts observed in many systems. Leading range edge populations, like those at upper elevation limits, are crucial for climate change responses but can exhibit low genetic diversity due to founder effects, isolation, or limited outbreeding. These factors can hamper local adaptation at range limits. Using the widespread herb, *Argentina anserina*, we measured ecological attributes (population density on the landscape, area of population occupancy, and plant and flower density) spanning a 1000 m elevation gradient, with high elevation populations at the range limit. We measured vegetative clonal potential in the greenhouse for populations spanning the gradient. We combined these data with a ddRAD-seq dataset to test the hypotheses that high elevation populations would exhibit ecological and genomic signatures of leading range edge populations. We found that population density on the landscape declined towards the high elevation limit, as is expected towards range edges. However, plant density was elevated within edge populations. In the greenhouse, high elevation plants exhibited stronger clonal potential than low elevation plants, likely explaining increased plant density in the field. Phylogeographic analysis supported more recent colonization of high elevation populations which were also more genetically isolated, had more extreme heterozygote excess and had smaller effective population size than low. Results support that colonization of high elevations was likely accompanied by increased asexuality, contributing to a decline in effective population size. Despite high plant density in leading edge populations, their small effective size, isolation and clonality could constrain adaptive potential.

## Introduction

Climate change has elicited rapid geographic range shifts (Chen et al. 2011) with the most common being upward in elevation (Klanderud and Birks 2003; Lenoir et al. 2008; Morueta-Holme et al. 2015; Freeman et al. 2018; Zu et al. 2021) and poleward in latitude (Chen et al. 2011). Range shifts may however be hampered in organisms with limited dispersal ability (Dullinger et al. 2012; Carnicero et al. 2022), and the inability to track climate change may lead to extirpation of popualtions if phenotypic plasticity or adaptation fail to buffer against environmental change (Hampe and Petit 2005). As genetic diversity is crucial for adaptive responses to enviromental change (Williams et al. 2008), understanding the distribution of genetic diversity and the population connectivity across species ranges offers insight into the capacity for populations to endure climate change, especially at environmentally stochastic range edges (Razgour et al. 2013; Rehm et al. 2015; Hargreaves and Eckert 2019; Angert et al. 2020; Sánchez‐Castro et al. 2022).

The distribution of genetic diversity across a species’ range is often influenced by a combination of factors including historical colonization, environmental suitability, and reproductive mode. Populations near the rear range edge, that is, those at lower elevation or latitude (also referred to as ‘warm edge’ or ‘trailing edge’) often served as a source of genetic material for the rest of the range following historical glaciations (Hampe and Petit 2005; Provan and Maggs 2012; Koski et al. 2019). Leading edge populations are often those at higher elevation or latitude that have been established more recently, which is frequently accompanied by a reduction in genetic diversity and increased differentiation as a consequence of founder effects (Nei et al. 1975; Pujol and Pannell 2008; González-Martínez et al. 2017; Koski et al. 2019). Thus, rear edge populations frequently harbor high genetic diversity, with declines in diversity towards the leading range edge (Alexandrino et al. 2000; Comps et al. 2001; Widmer 2001; Obbard et al. 2006; Griffin and Willi 2014). Reduced diversity and high genetic load in leading edge populations can reduce overall population performance (Bontrager et al. 2021). Gene flow from populations at the range core to the leading edge has long been theorized to limit local adaptation and limit range expansion (Kirkpatrick and Barton 1997; Fedorka et al. 2012; Angert et al. 2020; however see Kottler et al. 2021). In contrast however, such gene flow has recently been shown to benefit edge populations under extreme conditions associated with climate change (Bontrager and Angert 2019). Understanding how genetically diverse and isolated leading edge populations are is thus crucial for predicting whether they have the capacity to respond to climatic change via local adaptation or tracking suitable conditions via range expansion.

Ecological variation across ranges can also contribute to geographic patterns of genetic diversity. In general, marginal populations, either leading or rear edge, are expected to occupy less suitable environmental conditions relative to populations near the core, a tenet of the ‘abundant center hypothesis’ (Lawton 1993; Hargreaves et al. 2014; Lee-Yaw et al. 2016). As an extension, edge populations are predicted to be smaller and harbor less genetic diversity relative to core populations which are commonly genetically diverse and well-connected due to high suitable habitat that supports many populations with substantial gene flow (Eckert et al. 2008; Willi et al. 2018; Gougherty et al. 2020). Linking spatial patterns in population genetic diversity with ecological attributes of populations (e.g., population density on the landscape, census population size) offers insight into how ecological variation across ranges shapes population genetics.

In additional to historical colonization and ecological variation, reproductive mode can have a profound impact on patterns of genetic diversity across species’ ranges. The ability to colonize and establish new populations depends on life-history characteristics, dispersal mode, and reproductive mode (Angert et al. 2011; Pannell et al. 2015). For instance, plants that are able to self-fertilize or reproduce clonally may be favored during the process of establishment beyond range edges (Pannell and Barrett 1998). Limited sexual reproduction in clonal organisms (Silvertown 2008; Beatty et al. 2008; Arriesgado et al. 2015), and elevated selfing in those that are self-compatible (Koski et al. 2019), have a strong influence on demography and population genetics (Halkett et al. 2005). High levels of clonal reproduction for example, drives excess heterozygosity within populations (strongly negative F_IS_; Balloux et al. 2003; Halkett et al. 2005; Meloni et al. 2015; Stoeckel et al. 2006), but can have negative long-term repercussions for population persistence (Meloni et al. 2015). While individuals in leading edge populations may be well-equipped to colonize new populations due to reproductive assurance (Hargreaves and Eckert 2014), reduced genetic diversity associated with limited outbreeding could impede local adaptation (Hartfield and Glémin 2016).

In montane regions, widespread species often occur across steep ecological gradients with high elevation populations likely residing at the leading range edge. Among-population genetic differentiation is often strongly affected by position along elevation gradients (Ohsawa and Ide 2008; Reis et al. 2015; Polato et al. 2017). For instance, declines in population density on the landscape at high elevation can result in elevational reductions in population connectivity (Halbritter et al. 2019). Additionally, impediments to gene flow may change with elevation due to topographical barriers, establishing elevational patterns in isolation (Robin et al. 2015). Indeed, high elevation ‘sky island’ populations frequently exhibit strong genetic isolation (DeChaine and Martin 2005; Vásquez et al. 2016).

Elevational position along gradients can also shape patterns of within-population genetic diversity. Attributes of populations (size, density, distribution of individuals within populations) may vary with elevation if suitable habitat is unevenly distributed across elevation (Sagarin et al. 2006). In plants, the density and distribution of individuals within populations impacts pollen-mediated gene flow (Loveless and Hamrick 1984; Van Treuren et al. 1993; Richards et al. 1999; Franceschinelli and Bawa 2000), genetic diversity (Van Rossum et al. 2004), and the level of inbreeding (Coates and Sokolowski 1992; Tarayre and Thompson 1997). A review of plant population genetic diversity across elevation gradients revealed inconsistent patterns among taxa, with only 19% of studies supporting reduced genetic diversity at higher elevation (Ohsawa and Ide 2008). The drivers of the observed patterns however, were species-specific, but in most cases were not examined (Ohsawa and Ide 2008). Thus, it is important to obtain landscape- and population-level ecological data to contextualize the observed patterns of genetic diversity across elevation gradients.

*Argentina anserina* (Rosaceae) is a self-incompatible perennial herb with the ability to spread clonally via above ground stolons (herafter, runners). It is widespread in temperate regions in the Northern Hemisphere, where it spans wide elevation gradients in montane regions. We focused on 13 populations spanning >1000 m in Southwestern Colorado, with the highest elevation populations at the high elevational limit of the species range. We linked ecological attributes of populations (hereafter population attributes) that influence the genetic diversity and connectivity (population density on the landscape, plant distribution within populations, and clonal potential) with metrics of population genetic diversity to address the following questions and predictions:

1. How do population density on the landscape, and plant distributions within populations change from lower elevation populations to the high elevation range edge? We predicted that population density on the landscape should decline towards the edge, and that high elevation populations will occupy smaller areas, and have lower plant density than lower elevation populations.
2. Does the capacity for vegetative clonality exhibit an elevational cline? We predicted that if reproductive assurance is favored in leading edge populations, then vegetative clonality should increase with elevation.
3. Are populations genetically structured by elevation, and are high elevation populations more genetically isolated? We predicted that high elevation populations should be less admixed, more recently colonized, and more genetically isolated than low.
4. Do metrics of within-population genetic diversity (H_E,_ F_IS,_ N_E_) covary with elevation, and directly with any population attributes? We predicted that high elevation populations should have lower N_E_ and reduced H_E_ due to recent founder events, and more extreme heterozygote excess (negative F_IS_) if they are more vegetatively clonal.

## Materials and methods

### Species and location of study

*Argentina anserina* (L.) Rydb. (Rosaceae) is a perennial herb, with a cosmopolitan global distribution in temperate environments. It reproduces both sexually via self-incompatible flowers (Cisternas-Fuentes et al. 2023), and vegetatively via runners (Eriksson 1988). Flowers of *anserina* are predominately pollinated by small solitary bees and flies (Koski and Ashman 2015). In Colorado, *Argentina anserina* grows naturally on pond and river edges, and in wet meadows, but also occupies disturbed habitats like roadsides, grazing lands, and airfields. In the San Juan Mountains of Southwestern Colorado, population occur between 1900–3500 m.a.s.l.

**Fig. 1:**
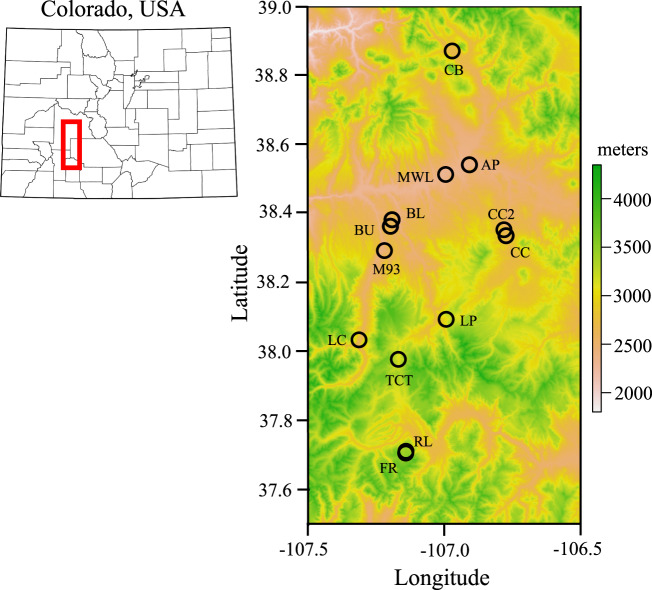
Focal populations of *Argentina anserina* across an elevational gradient in Southwestern Colorado. Map of the state of Colorado (left) indicating in red the location of the focal populations.

We studied 13 focal populations spanning over 1000 m to capture the majority of the elevation range occupied by *A. anserina* in SW Colorado (Fig. 1). The lowest elevation populations were in the Gunnison River Valley (~2300 m.a.s.l) and the highest elevation populations were at edges of high elevation kettle ponds or streams in the San Juan Mountains (~3400 m.a.s.l).

### Estimating population density across the elevation gradient

We used a collections-based approach to address whether the density of herbarium accessions of *A. anserina* changed with elevation in our sampling region (Question 1). Specifically, we measured herbarium accession density within each 100 m elevational band within our sample region. We first downloaded all herbarium accessions of *Argentina anserina* from the SEINet database, an online portal for herbarium specimens throughout the Western USA. We identified all specimens in our sampling area within a bounding box of 1.5 degrees latitude (37.5 to 39.0) and one degree longitude (−107.5 to −106.5) (Fig. 1). After removing duplicate accessions, our dataset included 79 unique accessions with elevation (m.a.s.l) data. Spatial sampling bias is pervasive in herbaria, including elevational sampling bias (Daru et al. 2018). To account for potential sampling bias we divided the number of *A. anserina* accessions within each 100 m elevational band by the number of herbarium accessions of all species (barring *A. anserina*) from SEINet with records of elevation. We again eliminated duplicate accessions prior to enumarting the number of non-focal taxa per 100 m elevational band. Finally, we downloaded a digital elevation model (DEM) raster of our sample area from the United States Geological Survey (USGS, https://apps.nationalmap.gov/downloader/#/). Using the ‘raster’ package in R (Hijmans and van Etten 2012) we estimated the total area (km^2^) within each 100 m elevational band between 2200 and 3500 m, roughly the extent of our elevational sampling. We calculated a metric of standardized population density of *A. anserina* for each 100 m band as: *A anserina* accessions */* total non-focal accessions / km^2^. We evaluated the relationship between standardized population density and elevation using a linear model in R (‘lm’) treating each elevation band quantitatively using its midpoint elevation.

### Classifying area of occupancy, density, and patchiness

To assess how plant and flower distributions within populations changed over the elvation gradient (Question 1) we measured the following attributes in each population in Summer 2021: area of occupancy, plant density, plant patchiness, flower density and flower patchiness. Together, these estimate the census size of the population, the density and distribution of plants within populations, and the density and distribution of sexually reproductive units within populations. During peak flowering (late June- early July 2021) we estimated the area of occupancy (area occupied by plants in m^2^) by walking straight-line transects and identifying population perimeters where plants were no longer present. We then ran two straight-line 50 m transects within each population, and within a 1x1m plot we scored the percent cover of *A. anserina* and the number of open flowers every 10 m (*n* = 10 plots per population). Plot locations were chosen randomly every 10 m by tossing a 1 × 1 m PVC-pipe square into the population.

We calculated population-level plant density as the average percent groundcover of *A. anserina* across all plots within a population, and flower density as the average number of flowers across plots within a population. We estimated plant patchiness as the coefficient of variation (CV) of *A. anserina* density across the 10 plots, and flower patchiness as the CV of flower number per plot. Populations with higher CVs for plant density and flower number among plots were more patchily distributed.

### Vegetatively clonal potential

To address whether clonal potential changed with elevation (Question 2), in 11 of the focal populations, we estimated metrics of vegetative clonal growth on plants in a common greenhouse environment. Plants from each population were collected 2^+^m from one another in the field in 2019, 2020, or 2021 and potted in a standard soil mix (3:1 Fafard to Turface). Each year, plants were subjected to a vernalization period in an environmental cold room set to 4.4 °C with total darkness for ~6 weeks once in the winter and summer. For 4 months in the Spring and 4 months in the fall, plants were kept in the greenhouse set to 15 °C. Plants were fertilized using slow-release Osmocote fertilizer (15/15/15, N/P/K) after each vernalization period. At the end of the plant growth cycle in Spring 2022, we measured the number of runners, the length of each runner, and the number of plantlets on each runner for 3–11 plants per population (mean = 7.2 + /−2.9 SD). In total we measured 280 runners across all plants. We calculated average runner length, average number of plantlets per runner, and plantlets per unit length of runner (cm) for each plant. From these metrics we generated population-level averages.

### DNA extraction and ddRADseq

We collected leaf tissue from natural populations or greenhouse material to obtain high-quality genomic DNA (20 ng/μL for a final amount of 1 μg). In each population, tissue from an individual was sampled at least every 2 m to reduce the likelihood of sampling ramets of the same genet. We selected 7–8 individuals per the 13 focal population (*N* = 95) and extracted total genomic DNA using a modified cetyltrimethylammonium bromide extraction protocol (CTAB; Doyle and Doyle 1987). DNA concentration was evaluated using Qubit HS and quality was evaluated in 1.5% agarose gel. Samples were prepared into a ddRAdSeq library with PstI and MseI restriction enzymes, and each sample was identified by a unique 11 bp sequenced (6 bp of barcode and 5 bp of Ilumina primers). The library was prepared and sequenced using llumina HiSeq by Floragenex, Inc (Portland, Oregon, USA). Each sample was sequenced on two separate lanes to maximize the number of reads and depth of the loci. Sequences from both lanes were combined before further analysis. We obtained over 570 million reads, and after removing reads without full barcodes, those without the enzyme cut site, and those with low-quality, we retained nearly 91% of the original reads.

To analyze the short read sequencing data, we used Stacks version 2.53 (Catchen et al. 2013). We used the de novo approach of the pipeline using the following parameters to maximize SNP recovery: M = 4 and m = 3 (ustacks), *n* = 2 (cstacks), *p* = 7, and *r* = 0.7 (populations). Different combinations of ustacks and populations parameters were tested before selecting parameters. Samples with a low number of reads retained (<60,000) following ustacks were eliminated from the dataset. Two samples from each population with the highest number of reads were used to create the catalog and a single SNP per locus was obtained from the data (–write-single-snp function).

On average 1,546,642 reads were aligned into putative loci after using the USTACKS step of the STACKS pipeline. After filtering for loci present in 70% of individuals per population and present in at least seven populations, the average number of loci retained was 482,425, and ranged from 299,852 to 608,715 across populations. Using the filtering parameters described above, a total of 5218 SNPs were variable across populations and this matrix was used to evaluate within and between population genetic diversity.

### Population genetic structure

To infer discrete population genetic structure and degree of admixture across the elvation gradient (Question 3), we ran conStruct analysis (Bradburd et al. 2018), which is similar to the Bayesian clustering analysis STRUCTURE (Pritchard et al. 2000), but accounts for isolation-by-distance by introduction of spatial layers defined by the value K. Specifically, allele frequence covariance decays with increasing spatial distance between populations. The original SNP matrix contained too many missing loci for model convergence in conStruct. Therefore, we generated a more restrictive matrix of 1081 SNPs with the following STACKS parameters: *p* = 10, and *r* = 0.8 (populations) to reduce the numer of missing SNPs across individuals. However, 6 indiviuals with >30% missing SNPs were removed from the dataset due to convergence issues. Each individual removed was from a separate population, thus removal was not biased. With this pruned matrix, we modeled population genetic structure with 1 to 13 layers with 1000 iterations per MCMC chain. We measured the relative contribution of each additional layer to total covariance. Layer contributions ranged 4 to 77% with an average of 30% (+/− 7% SE; Supplementary Fig. 1). We accepted any layer that contributed 30% or more to total covariance when added (layers 3–6, and 8). Layers 4 and 6 contributed the most to covariance (77%, and 64%, respectively) and were considered the best models.

One low elevation population, MWL, was an outlier for F_IS_ based on Grubb’s Outlier Test (G = 2.407, *P* = 0.034), an outlier for F_ST_ among the lower elevation populations (G = 2.609, *P* < 0.0001), and grouped genetically with high elevation populations in conStruct. This population is adjacent to a wastewater treatment plant, occurs on mounds of fill-dirt, and may be a very recent introduction. For these reasons, we excluded it from downstream analyses testing for elevational clines in population genetic parameters as well as ecological associations with genetic parameters.

Visual assessment of conStruct figures suggested that individuals in high elevation populations were less admixed than those in low. To evaluate how the magnitude of admixture varied along the elevation gradient in a quantitatve manner, we identified the highest cluster assignment probability for each individual using 4 and 6 layers/clusters. For example, in a model with 4 clusters/layers, an individual that was highly admixed would have 25% ancestry from each cluster, and a highest membership of 25%. Conversely, an individual assigned to a single cluster would have 100% membership. We then calculated a ‘cluster uniformity’ index for each population as the average of the highest layer membership across individuals, with higher values indicating lower admixture. We modeled both the cluster uniformity index as a function of elevation with the best models (layers 4 and 6).

### Population-level phylogeny

Phylogeograhpic approaches are commonly used to evaluate the impacts of historical colonization on contemporary genetic structure of populations (e.g., Hewitt 2000; Barnard-Kubow et al. 2015; Prior et al. 2020). If high elevation populations were colonized by founders from lower elevations, divergence time estimates should become more recent with increasing elevation (Question 3). We constructed a population-level phylogeny to test this prediction using a matrix of SNPs that were fixed within but variable between populations generated using –phylip in STACKS (Barnard-Kubow et al. 2015). This generated a population-level matrix of 11,507 sites. In RAxML (v. 2.0.10)(Stamatakis 2014), we determined that the TVM substitution model was the most appropriate based on AIC and AICc criterion from ModelTest-NG (v.0.1.7). We then generated 50 random start trees assuming a TVM substitution model and selected the best tree based on -lnL score. We generated bootstrap values from 200 trees. We used node age at the time of divergence to estimate each population’s divergence time. Because the phylogeny was not dated, we assumed that substitution rates are proportional to time.

### Isolation by lateral, vertical, and topographic distance

We estimated genetic isolation by distance by performing three Mantel tests using different metrics of distance between populations: geographic distance (lateral), vertical distance (elevation), and topographic distance which incorporates both lateral and vertical distance. We first tested for isolation by lateral distance using a Mantel test between matrices of pairwise F_ST_ and pairwise geographic distance (km). We calculated pairwise vertical distances (m) between populations as the absolute difference in elevation and conducted Mantel’s test for isolation by vertical difference. We tested for isolation by topographic distance by estimating least-cost paths between populations based on topography. Because gene flow between populations via pollen or seed is likely inhibited not only by distance but topography, accounting for topography has the potential to provide a better metric of functional distance between populations. Using the DEM raster of our sampling area, we plotted each of the 12 populations and calculated paths between points using ‘topoDist’ (Wang 2020). From any given cell, we allowed movement in 8 cardinal directions to reach the next cell when establishing paths. We again conducted a Mantel’s test using the pairwise F_ST_ matrix and the topographic distance matrix. All Mantel Tests were performed using the mantel.rtest function in the package ade4 (Dray and Dufour 2007).

### Metrics of within and between-population genetic diversity

To evaluate elevational patterns in population genetic parameters (Questions 3 and 4), we calculated the following population genetic diversity parameters in GenAlEx (Peakall and Smouse 2012): mean number of alleles per locus (N), percentage of polymorphic loci (%P), number of different alleles (NA), number of effective alleles (NEA), observed and expected heterozygosity (HO and HE) and inbreeding coefficients (FIS). We estimated effective population size (N_E_) using the heterozygote excess method in NeEstimator v. 2.1 (Do et al. 2014). This approach estimates the effective number of breeders, a proxy for N_E_, based on the observation that heterozygosity in progeny is greater than expected under Hardy–Weinberg Equilibrium when the number of breeders is small (Rasmussen 1979; Zhdanova and Pudovkin 2008; Gilbert and Whitlock 2015; Waples et al. 2016). In one population (AP), N_E_ was estimated as infinite. For this population, we assigned a conservative estimate of N_E_ using the highest N_E_ value observed among our populations (N_E_ = 15). We calculated pairwise F_ST_ in GenoDive v.3.0 (Meirmans 2020).

### Population and genetic attributes across the elevation gradient

To determine whether population attributes varied with elevation, we tested for elevational patterns using linear models in the following population attributes: area of occupancy, plant density, plant patchiness, flower density, and flower patchiness.We also modeled each metric of clonal potential measured in the greenhouse (number of runners, length of runners, plantlets per cm) as a function of elevation. We included the duration in years (1, 2, or 3) that a population persisted in greenhouse conditions as a covariate because being pot-bound could impact on clonal potential. To obtain the direct effect of elevation on clonality metrics alone, we generated residual clonality metrics at the population from a model of each as a function of year in the greenhouse. We then regressed residual clonality metrics from these models as a function of elevation using linear models.

We then tested whether H_E_, F_IS_, and F_ST_ exhibited clinal variation with elevation by modeling each a function of elevation using linear regression. Finally, using the population-level phylogeny, we extracted divergence times estimated at each node, and modeled divergence time as a function of elevation and longitude with bootstrap node support as a weighting factor. We included longitude as a covariate in the model because preliminary graphical evaluations showed that divergence time was associated with longitude. While our sampling spanned a narrow longitudinal range, waterways in the Gunnison River Basin flow east-to-west, potentially structuring population genetic diversity of *A. anserina* which is a primarily wetland species. We estimated standardized regression parameters using the ‘lm.beta’ function for elevation and longitude such that their effects on divergence time were directly comparable.

### Associating population attributes with heterozygosity

Because variation in heterozygosity among populations was not explained by elevation (see results), we tested whether H_E_ was directly predicted by population attributes measured in the field. To do so, we used stepwise regression starting with a global model predicting variation in H_E_ using the following: area of occupancy, plant density, plant patchiness, flower density and flower patchiness. We used the ‘step’ function in R to iteratively remove terms until reaching a model from which AIC values no longer declined significantly with additional deletion of a single predictor. For the final model, we evaluated the variance inflation factor (VIF) using the ‘vif’ function. The final model’s VIFs ranged 1.3–2.5, indicating minimal effects of multicollinearity (Sheather 2009). We additionally estimated standardized regression parameters using the ‘lm.beta’ function for each predictor variable such that their effects were directly comparable.

## Results

### Population attributes across the elevation gradient

**Fig. 2:**
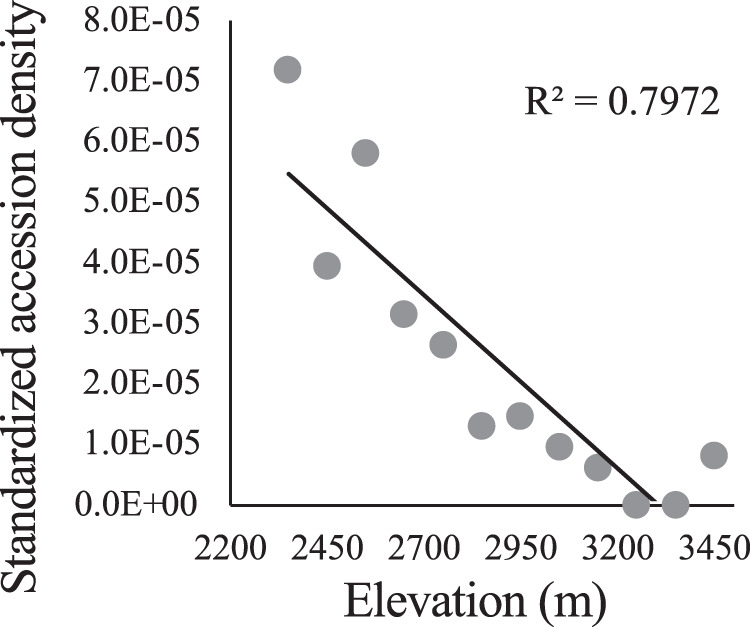
Number of herbarium accessions of *Argentina anserina* per km^2^ across the elevational gradient of focal populations (See Fig. 1). *Argentina anserina* accessions were standardized by total non-focal plant accessions in each 100 m elevation bin to account for potential collection bias.

**Fig. 3:**
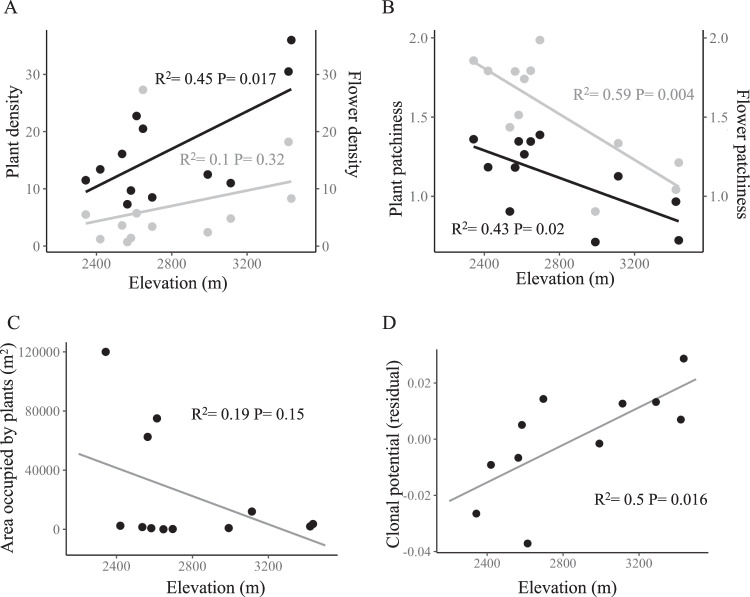
Elevational patterns of ecological attributes of *Argentina anserina* populations. **A** Plant density as percent cover (in black) and flower density as flowers per m (in gray), **B** plant and flower patchiness (in black and gray, respectively), **C** the area occupied by the population and **D** vegetative clonal potential measured as plantlets per cm of runner in a common garden. Residuals of clonal potential from a model accounting for the number of years that a population was in the greenhouse are plotted.

Accession density of *A. anserina* on the landscape declined precipitously with elevation (Fig. 2). The majority of population-level attributes also exhibited clinal variation with elevation. Plant and flower patchiness within populations show a decline with elevation (*R*^2^ = 0.43, *P* = 0.02; Fig. 3B; *R*^2^ = 0.59, *P* = 0.004; Fig. 3B). Area of occupancy tended to decline with elevation as well (*R*^2^ = 0.19, *P* = 0.15; Fig. 3C). Plant density within populations increased with elevation (*R*^2^ = 0.45, *P* = 0.017; Fig. 3A) while flower density was unaffected (*R*^2^ = 0.10, *P* = 0.32; Fig. 3A). Clonal potential evaluated as the number of plantlets per cm of runner measured in the greenhouse increased with elevation (R^2^ = 0.50, *P* = 0.016; Fig. 3D), while the number of runners (R^2^ = 0.027, *P* = 0.62) and the length of runners (R^2^ = 0.15, *P* = 0.24) did not show a strong association with elevation.

### Isolation by distance and elevation

Average between-population genetic differentiation across the 1000 m elevational gradient varied from 0.086 to 0.212 (mean F_ST_ = 0.13, SE = 0.012). Linear geographic distance between populations was a significant predictor of pairwise F_ST_ (Mantel’s Test *R*^2^ = 0.401, *P* = 0.03), while topographic distance was a weaker predictor of F_ST_ (Mantel’s Test *R* = 0.36, *P* = 0.054). The elevation difference between populations was the strongest predictor of F_ST_ (Mantel’s Test *R* = 0.65, *P* = 0.001) (Supplementary Fig. 2).

### Population genetic structure and phylogeography

**Fig. 4:**
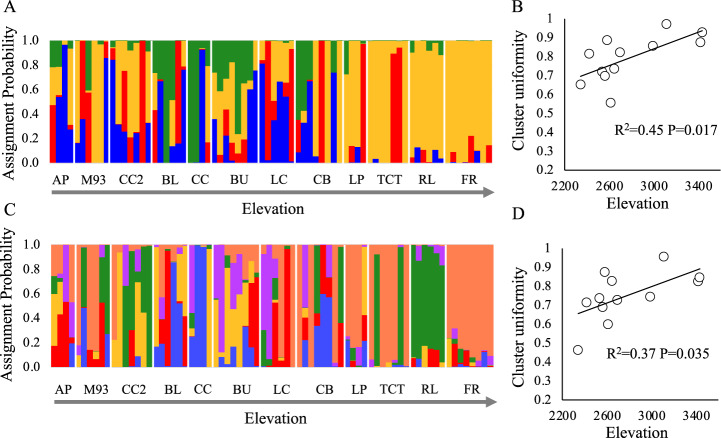
Population structure analysis of 12 *Argentina anserina* populations spanning >1000 m elevational gradient. Admixture barplots with **A** *K* = 4 and **C** *K* = 6. Populations are shown in order from lowest elevation to highest elevation (left to right). Panels **B** and **D** depict population-level average cluster uniformity plotted against elevation for *K* = 4 and *K* = 6, respectively. Higher uniformity indices indicate populations in which individuals are less admixed.

ConStruct analyses indicated that the additions of the fourth and sixth layers contributed most to covariance (Supplementary Fig. 1). With both K = 4 and K = 6, lower elevation populations exhibited high levels of admixture while higher elevation populations appeared more uniform in cluster assignment (Fig. 4A, C). A quantitative score of cluster uniformity at the population level indicated that individuals in high elevation populations were significantly less admixed than those in low elevation populations when K = 4 (*R*^*2*^ = 0.45, *P* = 0.017; Fig. 4B), and K = 6 *(R*^*2*^ = 0.37, *P* = 0.035, Fig. 4D).

**Fig. 5:**
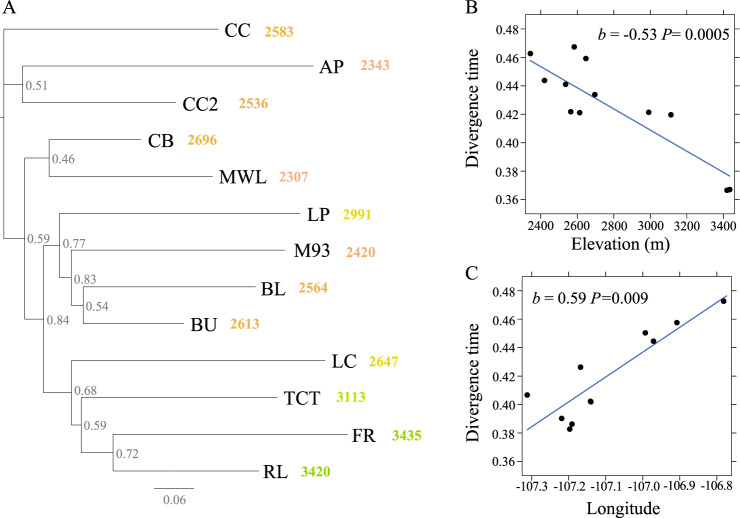
Population-level maximum-likelihood phylogeny of *Argentina anserina*. **A** Phylogeny based on SNPs fixed within but variable between populations from ddRAD-seq. An outgroup population was not assigned. Nodes indicate bootstrap support and branch lengths are untransformed. Population name and associated elevation (m) are provided at tips with colors corresponding to the elevation map in Fig. 1. The direct effects of elevation (**B**) and longitude (**C**) on divergence time estimates. Effects of elevation and longitude are from a model including both parameters that was weighted by the bootstrap support for each node (higher support = higher weight). The strong longitudinal effect likely reflects the east-west flow of waterways in of the Gunnison River Basin.

The population level phylogeny grouped three of the lowest elevation and most eastern populations (CC, AP, and CC2; Fig. 1) which had the earliest divergence time estimates (Fig. 5A). Low elevation populations (M93, BL, BU) formed a clade with one higher elevation population (LP) (Fig. 5A). The three highest elevation populations clustered (TCT, RL, FR) with a geographically proximal mid-elevation population (LC) (Fig. 5A). Finally, the most geographically isolated population in our sample (CB, Fig. 1) clustered with MWL (Fig. 5A). Across populations, 89% of the variation in divergence time was predicted by elevation and longitude (overall *P* = 0.0001) with both factors being important predictors (elevation, *P* = 0.005, longitude *P* = 0.009) (Fig. 5B, C). Divergence times of high elevation and more western populations were more recent than lower elevation and more eastern populations (Fig. 5B), and the magnitude of elevation and longitude effects were roughly the same (Fig. 5B, C).

### Elevational patterns of population genetic parameters

Within population genetic diversity metrics (%P, N_A_, H_O_, and H_E_) are provided in Table 1. H_E_ varied among populations over two-fold (0.05–0.13). The inbreeding coefficient (F_IS_) was consistently negative across populations, but exhibited wide variability from −0.14 to −0.78.

**Table 1:** Genetic diversity metrics in 13 populations of *Argentina anserina* spanning over 1000 m elevational gradient in Southwestern Colorado and the average across all populations.

Population	Elevation (m.a.s.l)	Sample size	*N*	%P	N_A_	N_EA_	H_O_	H_E_	F_IS_	F_ST_
MWL	2307	7	5.10	22.94	1.11	1.09	0.19	0.10	−0.78	0.212
AP	2343	5	1.90	18.24	0.65	0.58	0.07	0.06	−0.14	0.086
M93	2420	7	4.50	31.97	1.11	0.98	0.14	0.11	−0.20	0.109
CC2	2536	7	3.48	24.91	0.86	0.75	0.10	0.08	−0.14	0.097
BL	2564	7	2.88	20.66	0.72	0.64	0.09	0.07	−0.21	0.102
CC	2583	4	2.63	26.49	1.04	0.96	0.14	0.10	−0.34	0.105
BU	2613	8	6.33	39.36	1.28	1.09	0.16	0.12	−0.21	0.105
LC	2647	6	3.22	24.55	0.83	0.72	0.10	0.08	−0.16	0.106
CB	2696	8	6.65	38.56	1.29	1.12	0.17	0.13	−0.25	0.114
LP	2991	5	1.85	14.33	0.59	0.54	0.08	0.05	−0.36	0.131
TCT	3113	7	5.33	29.82	1.17	1.07	0.17	0.11	−0.39	0.169
RL	3420	7	5.10	23.25	1.09	1.04	0.17	0.10	−0.63	0.205
FR	3435	8	5.45	27.65	1.05	0.94	0.12	0.10	−0.22	0.177
All Populations (SE)		86	6.75 (0.01)	26.36 (2.01)	1.33 (0.002)	1.162 (0.002)	0.2 (0.001)	0.14 (0.001)	−0.296 (0.002)	0.132 (0.012)

**Fig. 6:**
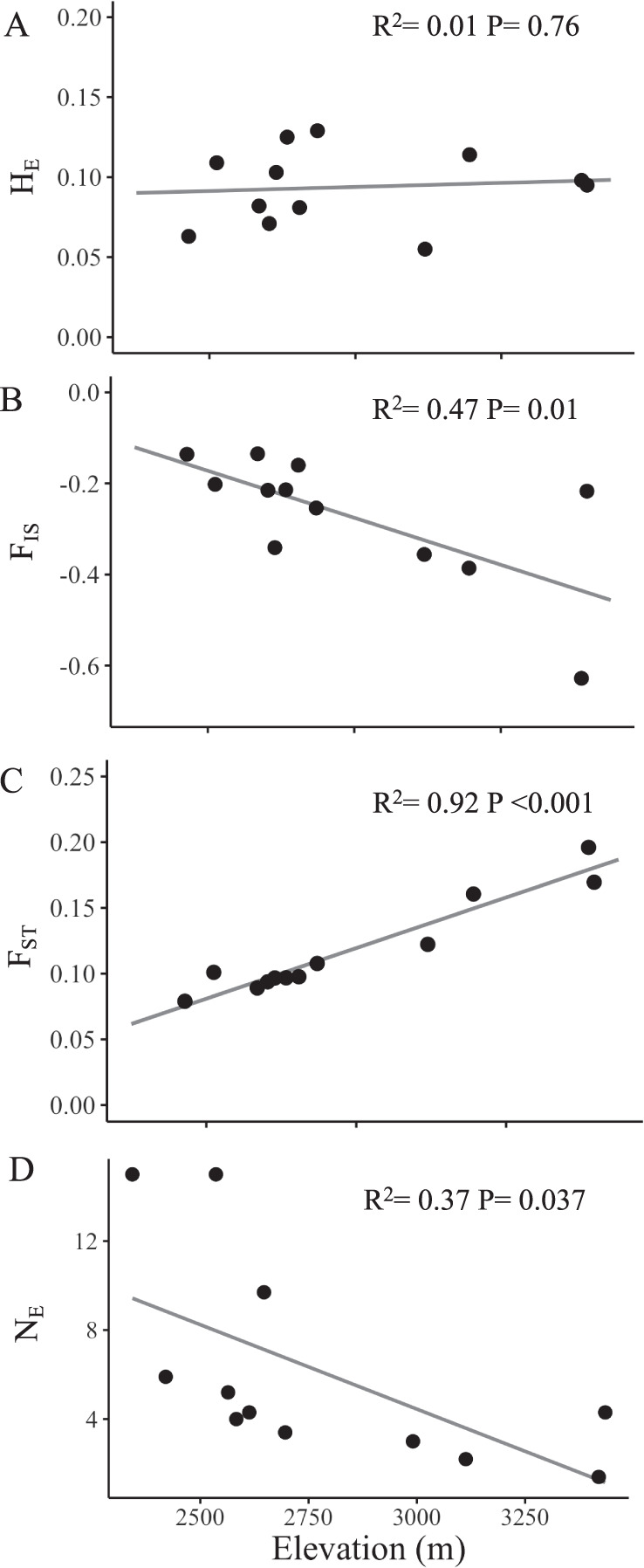
Elevational patterns of population genetic parameters. **A** H_E_, **B** F_IS_, **C** F_ST_ and **D** N_E_ plotted against elevation.

There were strong elevational patterns in F_IS,_ mean pairwise F_ST_, and N_E_. Specifically, F_IS_ became more negative with increasing elevation (*R*^2^ = 0.47, *P* = 0.014; Fig. 6B), indicating that high elevation populations harbor higher heterozygosity than expected under Hardy-Weinberg Equilibrium. Average pairwise F_ST_ increased with elevation (*R*^2^ = 0.92, *P* < 0.0001; Fig. 6C), indicating that higher elevation populations were more strongly isolated from others. N_E_ also declined significantly with elevation (*R*^2^ = 0.37, *P* = 0.037; Fig. 6D). Expected heterozygosity was unassociated with elevation (*R*^2^ = 0.01, *P* = 0.76; Fig. 6A).

### Links between population attributes and heterozygosity

Out of the five parameters included in the full model explaining variation in H_E_, four were retained in the best fit model determined by stepwise regression. Together, the area of occupancy, plant density, plant patchiness, and flower density explained 73% of the variation in H_E_ among populations (*P* = 0.037; Table 2). Higher H_E_ was associated with smaller area of occupancy, higher plant density, more patchy plant distribution within populations, and lower flower density (Table 2). Flower patchiness was not included in the final model based on model selection from stepwise regression.

**Table 2:** Results of linear model predicting variation in H_E_ based on population-level ecological attributes: area of occupancy, plant density, plant patchiness and flower density.

Effect	Estimate	SE	Standardized Estimate	*t*	*P*
Area of occupancy	−3.8 E-07	1.37 E-07	−0.642	−2.873	0.024
Plant density	0.003	0.001	1.011	3.225	0.015
Plant patchiness	0.112	0.028	1.151	4.050	0.005
Flower density	−0.002	0.001	−0.838	−2.950	0.021

The predictor variables in the model were chosen based on stepwise regression. Flower patchiness was not retained in the final model. Standardized estimates are provided such that the magnitude of each parameter’s effect on H_E_ are directly comparable.

Overall R^2^ = 0.73,

Overall *P* = 0.037.

## Discussion

Patterns of genetic diversity across elevation gradients can provide insight into both historical and contemporary processes impacting population structure at leading range edges. *Argentina anserina* populations at the high elevation range limit exhibited strong isolation, extreme heterozygote excess, and small effective population size. These patterns were associated with elevational reductions in population density on the landscape, increases in plant density within populations, and increased investment in clonal plantlets at high elevation. Strong isolation is common in populations at elevational limits of species ranges (e.g., Herrera and Bazaga 2008; Hahn et al. 2012; Sjölund et al. 2019), and heterozygote excess is consistent with scenarios of increased clonality (Balloux et al. 2003; Halkett et al. 2005; Stoeckel et al. 2006; Meloni et al. 2015), small effective population size (Balloux et al. 2003), and/or recent bottlenecks (Luikart 1998). Our data suggest that high elevation populations have likely experienced all three. Low effective population size, high clonality, and isolation at the leading edge in this system are all factors that could limit both adaptive responses to altered climate at or beyond contemporary range limits.

### Isolation, differentiation, and small effective population size at a high elevation range edge

The elevational cline in F_ST_, strong isolation by vertical distance, and conStruct analyses all supported that high elevation populations were more genetically isolated than lower elevation populations. Over 90% of the variation in F_ST_ among populations was explained by elevation with lower elevation populations being less isolated (F_ST_ ~ 0.10) and higher elevation populations exhibiting higher isolation (F_ST_ > 0.15). These increases in isolation are accompanied by a reduction in the density of *A. anserina* accessions at high elevations, which we interpret as a reflection of lower population density at higher elevations because we controlled for elevational variation in collection bias. Our results are consistent with increased isolation at high elevation being driven greater distances between extant populations. *Argentina anserina* occupies the edges of ponds and streams, as well as wet meadows and roadsides, but does not commonly occur on steep slopes. A more rugged topography at higher elevation in the San Juan Mountains could limit suitable habitat for this species.

Genetic isolation was better explained by vertical distance between populations than by lateral or topographical distance. This suggests that upward or downward elevational gene flow, either via pollen or seed, is likely constrained in this system. There was also more admixture among individuals in lower elevation populations based on conStruct analyses. When K = 6, even high elevation popualtions that are very geographically close to one another (RL and FR, ~660 m apart) were assigned to different clusters. This suggests that higher elevation populations are not only isolated from lower elevation populations, but from one another. Low connectivity of high elevation populations, especially from lower elevation populations that experience warmer conditions, has the potential to limit the introduction of novel genetic variants that could increase genetic diversity and contribute to enhanced performance under climate change (e.g., Bontrager and Angert 2019).

While contemporary gene flow into high elevation populations may be limited, phylogeographic evidence suggests that high elevation populations were likely founded by low-elevation sources. The population-level phylogeny placed three of the lower elevation populations near the base of the tree, while higher elevation populations formed a clade that diverged more recently. More recent divergence events are a signature of leading range edge populations (e.g., Prior et al. 2020). The linear decline in divergence time with elevation and with longitude suggest westward and upward colonization route of *A. anserina* in this region. The strong east-west pattern is likely driven by the westward flow of waterways in the Gunnison River Basin, as *A. anserina* is largely restricted to floodplains and river edges in this area.

### Clonality contributes to elevational patterns of population density and population genetic diversity

High elevation populations tended to occupy smaller areas than lower elevation populations but had higher plant and flower density. Additionally, within high elevation populations, plants were more continuously distributed within populations compared to low elevation, where plants were more patchily distributed. These elevational patterns may have arisen due to a combination of intrinsic attributes of the plants themselves, as well as environmental attributes that differ across the elevation gradient. First, we found that high elevation genotypes had higher clonal potential in a common garden than low elevation genotypes. Specifically, high elevation genotypes produced more plantlets per unit length of runner. While runner number and runner length did not show elevational patterns, production of more clonal plantlets per unit length of runner suggests increased investment in clonal propagules. High density and low patchiness could be the result of stronger investment in clones by plants at high elevation populations. The general increase in flower density at higher elevation seems counterintuitive if populations are more clonal. However, because flowers can be borne along runners at nodes with clonal plantlets, this pattern is still consistent with increased clonality. A second, non-mutually exclusive explanation of increased plant density at high elevation is a change in habitat conditions with elevation. Lower elevation populations tend to occur in wet floodplains, or densely vegetated willow thickets. The two highest elevation populations in our sample (RL and FR) occur on wet gravelly edges of kettle ponds with reduced competition for space from other plant species. Thus, opportunity for clonal spread may be higher in high elevation populations.

The potential for increased clonality in field conditions at high elevation was reflected in the population genetic data as well. Strongly negative F_IS_ (extreme heterozygote excess) is a common feature of highly vegetatively clonal plant populations (Balloux et al. 2003; Halkett et al. 2005). All populations exhibited negative F_IS_, indicating high outbreeding, which is expected in *A. anserina* since it has a gametophytic self-incompatible breeding system (Cisternas-Fuentes et al. 2023). However, higher elevation populations tended to have more negative F_IS,_ potentially due to establishment of few clonal genotypes that proliferated through asexual reproduction. If the elevational cline in F_IS_ is driven in part by a cline in clonality, we predict that stronger clonal potential measured in a common garden should be associated with more negative F_IS_. Indeed, a post-hoc analysis showed that populations producing more plantlets per runner had more negative F_IS_ indicating heterozygote excess (*b* = −0.106 + /−0.03; *R*^2^ = 0.60, *P* < 0.01).

Further support for the establishment by relatively few individuals at higher elevation comes from the pattern of low effective population size at high elevation. The second highest elevation population had an estimated N_E_ of only 1.4 individuals while two low elevation populations had an estimated N_E_ of 15 or more. The continuous decline in N_E_ with elevation is strongly suggestive of bottlenecks occurring as populations expanded from low to high elevation. Similar patterns of N_E_ have been observed in other species during historical colonization (Gomaa et al. 2011; Polato et al. 2017; Koski et al. 2019).

While we found elevational patterns for isolation, inbreeding coefficients, and effective population size, expected heterozygosity remained constant across the gradient. That is, leading edge populations harbored as much heterozygosity as low. A suite of population attributes were important for explaining over 70% of the variation in heterozygosity among populations, though none of these factors changed consistently with elevation. In particular, populations occupying larger areas (characteristic of lower elevations) and those with higher flower density (characteristic of higher elevation) were associated with higher heterozygosity. Likewise, populations with more patchily distributed plants (low elevation) and those with higher plant density (high elevation) were linked with higher heterozygosity. Together, these correlates with heterozygosity were inconsistent with elevational patterns in population attributes, nullifying elevational trends in heterozygosity. If most heterozygosity in high elevation populations is harbored within individuals that largely propagate via vegetative clonality however, the generation of novel genotypes through sexual reproduction is unlikely.

## Conclusions

Our study revealed a suite of ecological attributes that correlate with a decline in connectivity and effective population size towards the high elevation range limit of *Argentina anserina* in the Southern Colorado Rocky Mountains. Isolation of high elevation populations likely reflects limited dispersal across elevation bands as well as a reduction in the frequency of populations at high elevation. Increased vegetative clonality near the upper range limit likely contributes to reductions in effective population size, increased heterozygote excess, and increased plant density. While high elevation populations appear large due to high plant density, the fact that they are effectively small, highly clonal, and isolated suggests that they could have limited adaptive potential in response to altered environmental conditions which have been pronounced at high elevation populations in Western North America (Diaz and Eischeid 2007).

## Supplementary information

Supplementary material

## Data Availability

All data and scripts used to tun the analysis are available at Dryad at 10.5061/dryad.5tb2rbp8h.
