# Supplementary material for: Drivers of strong isolation and small effective population size at a leading range edge of a widespread plant
Source: Heredity (Edinb). 2023 Apr 4;130(6):347–57. doi: 10.1038/s41437-023-00610-z (PMC10238488; doi:10.1038/s41437-023-00610-z)
Supplement: Supplementary file 1 — Supplementary material [file 41437_2023_610_MOESM1_ESM.docx]

**Drivers of strong isolation and small effective population size at a leading range edge of a widespread plant**

Anita Cisternas-Fuentes and Matthew Koski

Supplementary Fig. 1

Relative contribution of each additional layer to total covariance of conStruct analysis. Layers 4 and 6 contributed the most to covariance and were considered the best models.

Supplementary Fig. 2

Isolation by distance (A), isolation by topography (B) and isolation by elevation (C)
